# Impact of the Interaction between 3′-UTR SNPs and microRNA on the Expression of Human Xenobiotic Metabolism Enzyme and Transporter Genes

**DOI:** 10.3389/fgene.2012.00248

**Published:** 2012-11-21

**Authors:** Rongrong Wei, Fan Yang, Thomas J. Urban, Lang Li, Naga Chalasani, David A. Flockhart, Wanqing Liu

**Affiliations:** ^1^Department of Medicinal Chemistry and Molecular Pharmacology, College of Pharmacy, Purdue UniversityWest Lafayette, IN, USA; ^2^Center for Human Genome Variation, School of Medicine, Duke UniversityDurham, NC, USA; ^3^Division of Medical and Molecular Genetics, School of Medicine, Indiana UniversityIndianapolis, IN, USA; ^4^Division of Gastroenterology and Hepatology, School of Medicine, Indiana UniversityIndianapolis, IN, USA; ^5^Division of Clinical Pharmacology, Department of Medicine, Indiana University School of MedicineIndianapolis, IN, USA

**Keywords:** eQTL, xenobiotic metabolism enzyme and transporter, microRNA, pharmacogenetics, 3′-UTR

## Abstract

Genetic variation in the expression of human xenobiotic metabolism enzymes and transporters (XMETs) leads to inter-individual variability in metabolism of therapeutic agents as well as differed susceptibility to various diseases. Recent expression quantitative traits loci (eQTL) mapping in a few human cells/tissues have identified a number of single nucleotide polymorphisms (SNPs) significantly associated with mRNA expression of many XMET genes. These eQTLs are therefore important candidate markers for pharmacogenetic studies. However, questions remain about whether these SNPs are causative and in what mechanism these SNPs may function. Given the important role of microRNAs (miRs) in gene transcription regulation, we hypothesize that those eQTLs or their proxies in strong linkage disequilibrium (LD) altering miR targeting are likely causative SNPs affecting gene expression. The aim of this study is to identify eQTLs potentially regulating major XMETs via interference with miR targeting. To this end, we performed a genome-wide screening for eQTLs for 409 genes encoding major drug metabolism enzymes, transporters and transcription factors, in publically available eQTL datasets generated from the HapMap lymphoblastoid cell lines and human liver and brain tissue. As a result, 308 eQTLs significantly (*p* < 10^−5^) associated with mRNA expression of 101 genes were identified. We further identified 7,869 SNPs in strong LD (*r*^2^ ≥ 0.8) with these eQTLs using the 1,000 Genome SNP data. Among these 8,177 SNPs, 27 are located in the 3′-UTR of 14 genes. Using two algorithms predicting miR-SNP interaction, we found that almost all these SNPs (26 out of 27) were predicted to create, abolish, or change the target site for miRs in both algorithms. Many of these miRs were also expressed in the same tissue that the eQTL were identified. Our study provides a strong rationale for continued investigation for the functions of these eQTLs in pharmacogenetic settings.

## Introduction

Xenobiotic metabolizing enzymes and transporters (XMETs) are involved in biotransformation and detoxification of carcinogens, environmental toxins, and therapeutic drugs (Carlsten et al., 2008; Korkina et al., 2009). In humans, the process of biotransformation and detoxification of xenobiotics by XMETs can be divided into three phases: modification (phase I) primarily by enzymes of the cytochromes P450 superfamily; conjugation (phase II), e.g., glucuronidation by UDP-glucuronosyl transferase; and excretion (phase III) mainly by membrane transporters. XMETs are expressed in almost all tissue types, centrally and locally protecting the entire body against the damages caused by various natural and synthetic compounds. XMETs are highly expressed in digestive tract and especially in the liver, the most important organ for central metabolism (Conde-Vancells et al., 2010). Variations in the expression and activity of these XMETs lead to significant inter-individual difference in the disposition of exogenous chemicals including absorption, distribution, metabolism, and excretion (ADME) of pharmaceutical drugs. On the other hand, many XMETs are also found to be very abundant in non-digestive tract tissues/cells, e.g., brain, lung, bladder, and blood (Pavek and Dvorak, 2008). These XMETs could affect the local response to certain drugs at the site of action. Meanwhile, due to the crucial role of XMETs in detoxification of carcinogens and toxins, genetic variation in XMETs function in specific tissues/organs is also an important mechanism underlying genetic susceptibility to certain diseases, e.g., those XMETs expressed in lung and bladder may modify cancer risk. Recent genome-wide association studies have identified polymorphisms at the *UGT1A* locus strongly associated with urinary bladder cancer risk (Selinski et al., 2012). XMETs are sensitively regulated by various nuclear receptors (NRs) and transcription factors (TFs). These *trans*-acting regulators play a pivotal role in mediating cellular response to exposure to xenobiotics by modulating the transcription of XMETs, thus significantly contributing to the variability in the function of XMETs (Bourgine et al., 2012).

Identifying the DNA polymorphisms leading to the variations in XMET function is a major area of interest in pharmacogenetic and genomic research. To date, numerous studies focused on individual XMET genes have discovered a large number of sequence variations, many of which alter protein coding sequence and consequently affecting the activity of XMETs (Adjei et al., 2003; Hildebrandt et al., 2004; Ji et al., 2005; Moyer et al., 2007; Mrozikiewicz et al., 2011). Meanwhile, even more variants were suggested to quantitatively modulate gene transcription (Pavek and Dvorak, 2008). Recently, genome-wide mapping for gene expression quantitative trait loci (eQTLs) in a few human tissues/cells offered unprecedented opportunities to identify the most influential single nucleotide polymorphisms (SNPs) determining gene expression level of XMETs (Gamazon et al., 2010). However, unlike the variants located in the protein coding sequences for which the causality for altered enzyme activity can be more easily understood, how eQTLs affect gene transcription is largely unknown. Understanding the underlying mechanisms will lead to identification of novel causative DNA variants for XMET function as well as reliable pharmacogenetic markers.

MicroRNAs (miRs) are single stranded, about 22-nucleotides (nt) long, evolutionarily conserved, and function as important posttranscriptional regulators of mRNA expression by binding to the 3′-UTR of target mRNAs (Ambros, 2004; Bartel, 2004). MiRs are involved in various developmental and physiological processes by negatively regulating gene expression (Zhang et al., 2007). Over 30% of all protein-coding genes were estimated to be regulated by miRs (Brennecke et al., 2005; Krek et al., 2005; Lewis et al., 2005; Lim et al., 2005). Due to the conservation of the miR target site, SNPs located in 3′-UTR sequences may abolish or create a miR target, thus significantly affecting the mRNA expression (Saunders et al., 2007). Previous studies have suggested that many XMETs are regulated by miRs (Tsuchiya et al., 2006; Takagi et al., 2010; Patron et al., 2012). Several studies also demonstrated that SNPs in XMET gene 3′-UTRs led to different levels of enzyme activity (Saunders et al., 2007; Chin et al., 2008). Hence, we hypothesized that it may be an important mechanism that common SNPs or their linkage disequilibrium (LD) proxies located in the XMET gene 3′-UTR sequences alter mRNA expression via interference with miR targeting. In order to identify these candidate SNPs that may significantly modulate XMET expression, in this study we used multiple published human eQTL datasets to perform an *in silico* screening for SNPs that highly correlated with mRNA level of 409 major XMET genes. The significant SNPs and/or their LD proxies located in the gene 3′-UTRs were selected to predict a potential interference with miRs. We found that 27 SNPs located in the 3′-UTR of 14 XMET genes are likely associated with gene expression via altering miR binding.

## Materials and Methods

### Selection of eQTLs

**Figure 1:**
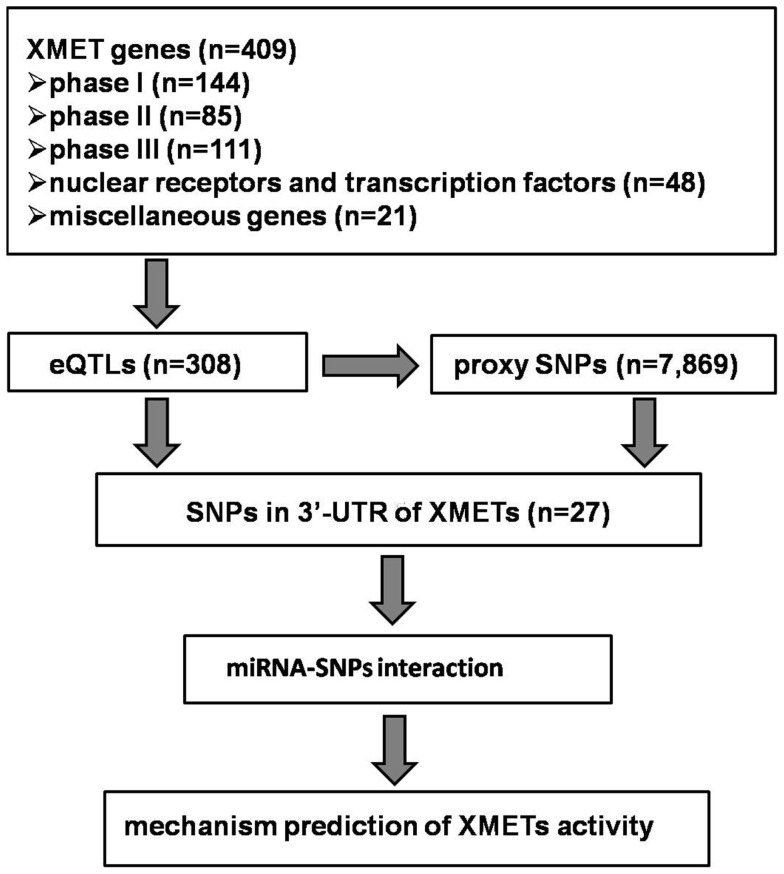
**Schematic of the search for miRNAs and the associated SNPs from XMET genes**.

The general strategy for the data analysis was presented in Figure 1. We used the published eQTLs datasets generated from the HapMap lymphoblastoid cell lines (LCLs; Montgomery et al., 2010), human liver (Schadt et al., 2008), and human brain (Gibbs et al., 2010). Although additional eQTL datasets in human LCLs are also available, we chose to use the one by Montgomery et al. (2010) which utilized high-throughput sequencing for the quantification of gene expression, as this technology has been suggested to produce more accurate gene expression data. To our knowledge, all datasets were collected from tissue/cells derived from individuals of Caucasian in origin. We used the online tool^1^ to search statistically significant eQTLs. As our study was focused on *cis*-acting eQTLs, we used a cut-off of *p* = 10^−5^ for significance, considering the window for genomic region (500 kb) of each gene and the potential number of SNPs (1 in every 100–1,000 bp).

### Search for SNPs in LD with eQTLs

To search SNPs in LD with significant eQTLs, we used the SNAP^2^ program to screen the 1,000 Genome SNP data within 500 kb range of the eQTLs of interest in the CEU population with a LD level cut-off of *R*^2^ = 0.8. Annotation for the location of eQTLs and their proxies relative to the gene structure was also collected with the program. Only SNPs and/or their proxies located within the 3′-UTR of the studied genes of interest were retained for further analyses.

### Prediction of SNP-miR interaction

In order to predict the potential SNP-miR interaction, two programs, MicroSNiPer^3^ and PolymiRTS^4^ were used. The major difference between the two programs is the algorithm used to predict the target site of miRs. The PolymiRTS program used the TargetScan^5^; Lewis et al., 2005; Friedman et al., 2009) algorithm (Bao et al., 2007). In contrast, the MicroSNiPer program used the FASTA (Pearson and Lipman, 1988) alignment program to determine if a change in a nucleotide in 3′-UTR sequence would change the miR binding capability, based on the requirement of perfect Watson–Crick match to the seed 2–7 nt of miRs (Lewis et al., 2005). To be conservative, we used 7-mers match as the cut-off value for a positive prediction.

## Results

### Genome-wide eQTL analysis of XMETs

**Figure 2:**
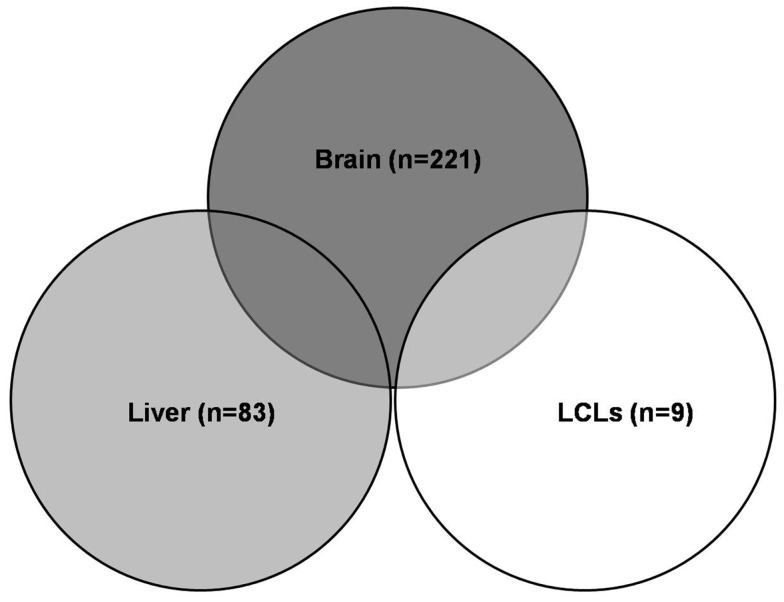
**Significant eQTLs in different tissues**. A total of 308 significant eQTLs were identified, including 9 eQTLs in LCL, 83 in liver, and 221 in brain tissues. Five eQTLs were shared in two tissue types.

Expression quantitative traits loci were screened for all 409 major XMET genes, including 144 phase I, 85 phase II and 111 phase III genes, 48 NRs, and transcription factor genes as well as another 21 genes related to drug ADME (Table A1 in Appendix). As a result, a total of 308 significant (*p* < 10^−5^) eQTLs were identified from 101 XMET genes. These include nine in LCL, 83 in liver, and 221 in brain tissues. Five SNPs were found as eQTLs shared in two tissue types: rs1023252 in both LCL and brain tissues, rs11101992, rs156697, rs2071474, and rs241440 in both liver and brain tissues (Figure 2). Among the total of 308 eQTLs, 20 SNPs were found to be located in the 3′-UTR region; 3 SNPs were in the 5′-UTRs; 171 SNPs were intronic; 8 and 6 SNPs were synonymous and non-synonymous coding variants, respectively; and 12 and 15 SNPs were located in the upstream and downstream flanking region of the genes, respectively. The remaining 73 SNPs were located in intergenic regions.

### eQTLs and their LD proxies

We chose to screen the 1,000 Genome SNP dataset as this would produce the most comprehensive coverage for the SNPs that may be in LD with a given eQTL. A total of 7,869 SNPs with significant LD with 260 eQTLs were identified. Combined with the remaining 48 eQTLs which had no reliable proxies in the 1,000 Genome dataset, a total of 8,177 SNPs (308 eQTLs and 7,869 proxy SNPs) were included in the subsequent analyses.

### Prediction of miR-SNPs interaction

Of the 112 eQTLs and proxies located in the 3′-UTR sequences, 27 SNPs were found in the 3′-UTR of 14 genes of interest. The remaining SNPs were located in nearby genes thus were excluded from the subsequent analysis. These SNPs were all common SNPs with their minor allele frequency (MAF) ≥0.067. Among the 27 SNPs, 12 were found in liver, and 15 were identified in brain tissue. More detailed information for these SNPs was listed in Table A2 in Appendix.

We focused our study on the association between miRs and these 27 SNPs in the 14 genes. After screened with the two algorithms, MicroSNiPer (Barenboim et al., 2010) and PolymiRTs (Gong et al., 2012), all the 27 SNPs apart from rs11807 (which is not predicted to be in a target site in PolymiRTs database) were found to potentially create, abolish, or alter the target site for miRs in both algorithms. Notably, 34 miRs were predicted by both algorithms to interact with 19 of these SNPs (Table A2 in Appendix). Of these 34 overlap miRs, except for rs2480256 of CYP2E1 which is not located in the seed sequence of hsa-miR-570-3p, all the remaining SNPs were found to be located in the seed sequence of miR targets.

To further validate the interaction between miRs and SNPs, we investigated whether the identified miRs were expressed in the same tissue as the identified eQTL. We used the GEO datasets (GSE21279 and GSE26545) to screen miR expression in liver and brain tissues, respectively (Hou et al., 2011; Hu et al., 2011). Since many predicted miRs were new and not probed by the published platforms, we thus only concentrate on the list of miRs probed in the platforms. Overall, over 74% (20 out of 27) of the identified miR-SNPs were found to have at least one predicted miR co-expressed with the gene of interest in the same tissue.

We further aimed to investigate whether these 27 SNPs are more likely to be targeted by miRs especially by the co-expressed miR in liver and brain tissues, compared to random-selected 3′-UTR SNPs with similar MAF. No statistical significance were found, possibly due to the limited power caused by the small number (*n* = 27) of SNPs involved (data not shown).

## Discussion

Although a large number of DNA variants affecting the function of XMETs have been identified, and many of them have been well linked with clinical response to pharmacotherapy or disease susceptibility (Motsinger-Reif et al., 2010), genetic variations in the activity of most XMETs remain incompletely explained. Recent studies continue to discover novel functional variants in XMET genes (Ramsey et al., 2012). Meanwhile, genome-wide association studies have found a number of XMET SNPs without previously known function significantly associated with different phenotypes in humans (Teichert et al., 2009; Estrada et al., 2012). These studies consistently suggested that additional sequence variants with fundamental role in XMET function have not been identified. Recent eQTL mapping in human tissues provided an opportunity to discover functional XMET polymorphisms at the genome-wide level. However, questions remain whether the identified eQTLs are causal for the altered gene expression and via what mechanism. Our study provides a comprehensive evaluation for this question in major human XMET genes, and generated a list of candidate SNPs that may modulate XMET genes via interference with miR targeting in multiple human tissue types.

Single nucleotide polymorphisms located in the gene 3′-UTRs could have great impact on miR targeting. It has been demonstrated that the entire 3′-UTR sequence could play important roles in miR function in addition to miR target sites (Hu and Bruno, 2011). In particular, negative selection in humans is stronger on computationally predicted conserved miR binding sites than on other conserved sequence motifs in 3′-UTRs, and polymorphisms in predicted miR binding sites are highly likely to be deleterious (Chen and Rajewsky, 2006). Gong et al. (2012) mapped SNPs to the 3′-UTRs of all human protein coding genes. Their results showed that among the 225,759 SNPs identified in 3′-UTRs, over 25% of SNPs potentially abolished 90,784 original miR target sites, while another 25% created a similar number of putative miRNA target sites. Besides these *in silico* studies, a number of SNPs altering miR targeting have been experimentally demonstrated to be associated with multiple diseases as well as drug metabolism and environmental procarcinogen detoxification (Abelson et al., 2005; Tan et al., 2007; Yu et al., 2007; Yokoi and Nakajima, 2011). Although the seed sequences for miR binding are critical and highly conserved, recent studies have also suggested that 3′-UTR sequences outside of the seed sequences, e.g., flanking sequences may be equally important for miR targeting by controlling the accessibility of the miR or local RNA structure (Grimson et al., 2007). For example, a SNP (829C > T) located 14 bp downstream of a miR-24 binding site in the 3′-UTR of human dihydrofolate reductase gene (*DHFR*) was demonstrated to affect *DHFR* expression by interfering with miR-24 function, resulting in *DHFR* over expression and methotrexate resistance (Mishra et al., 2007). By using two algorithms predicting potential SNP-miR interaction, we suggested that 27 eQTLs or their proxies in high LD for 14XMET genes may function through interference with one or more miRs, with most of the SNPs located in the seed sequences. Meanwhile, the majority (20 out of 27) of the identified miR-SNPs were found to have predicted miR co-expressed with the gene of interest in the same tissue. Although no statistically significant enrichment of miR targeting for these SNPs, the strong trends observed here warrants further experimental validations.

Our findings may also provide useful information in addition to the previous observations on the function of these SNPs. Previous studies demonstrated that SNP rs2480256 in the *CYP2E1* gene was significantly associated with systemic lupus erythematosus (Liao et al., 2011). Another study showed that cyclosporine A concentration in serum was significantly correlated with the genotype of the *CYP3A5* rs15524 polymorphism (Onizuka et al., 2011). In addition, a *GSTM3* haplotype including rs1537236 was significantly associated with a decreased growth for maximum mid-expiratory flow rate (MMEF) in a large population-based lung function study (Breton et al., 2009). SNP rs11807 in the 3′ region of *GSTM5* was found to be associated with hypertension (Delles et al., 2008). Our results thus may help further elucidate the mechanism(s) by which the SNPs are involved in the susceptibility to these specific phenotypes.

In conclusion, our study summarized the potentially interacting SNP-miRs that may affect the expression of major XMET gene, which may ultimately facilitate to elucidate the mechanism how these genes are regulated as well as how they are involved in the genetic variations in drug metabolism and disease pathogenesis. Further investigations are necessary to corroborate the hypotheses generated in this study.

## Conflict of Interest Statement

The authors declare that the research was conducted in the absence of any commercial or financial relationships that could be construed as a potential conflict of interest.

## Footnotes

^1^ http://www.ncbi.nlm.nih.gov/gtex/GTEX2/gtex.cgi

^2^ http://www.broadinstitute.org/mpg/snap/ldsearch.php

^3^ http://cbdb.nimh.nih.gov/microsniper

^4^ http://compbio.uthsc.edu/miRSNP/

^5^ http://www.TargetScan.org/

## Appendix

**Table A1:** **Major XMETs and related genes investigated in this study**.

Phase I (*n* = 144)	Phase II (*n* = 85)	Phase III (*n* = 111)	Nuclear receptors and transcription factors (*n* = 48)	Miscellaneous genes (*n* = 21)
AADAC	AANAT	ABC1	AHR	CRABP1
ABP1	ACSL1	ABCA1	AHRR	CRABP2
ADH1A	ACSL3	ABCA2	AIP	CYB5A
ADH1B	ACSL4	ABCA3	ARNT	GZMA
ADH1C	ACSM1	ABCA7	ARNT2	GZMB
ADH4	ACSM2B	ABCA8	CREBBP	MT1A
ADH5	ACSM3	ABCB1	EP300	MT1B
ADH6	AGXT	ABCB10	ESR1	MT1F
ADH7	AS3MT	ABCB11	ESR2	MT1H
ADHFE1	ASMT	ABCB4	FOXA2	MT1M
AKR1A1	BAAT	ABCB5	FOXO1	MT1X
AKR1B1	CCBL1	ABCB6	HIF1A	MT2A
AKR1B10	CES5A	ABCB7	HIF3A	MT3
AKR1C1	COMT	ABCB8	HNF4A	MT4
AKR1C2	DDOST	ABCB9	HSP90AA1	MTHFR
AKR1C3	GAMT	ABCC1	KEAP1	POR
AKR1C4	GGT1	ABCC10	NCOA1	RBP1
AKR1CL1	GLYAT	ABCC11	NCOA2	RBP2
AKR1D1	GNMT	ABCC12	NCOA3	TP53
AKR1E2	GSTA1	ABCC12	NCOR1	TXN
AKR7A2	GSTA2	ABCC2	NCOR2	TXN2
AKR7A3	GSTA3	ABCC3	NFE2L2	
AKR7L	GSTA4	ABCC4	NR0B2	
ALDH16A1	GSTA5	ABCC5	NR1H2	
ALDH18A1	GSTK1	ABCC6	NR1H3	
ALDH1A1	GSTM1	ABCC8	NR1H4	
ALDH1A2	GSTM2	ABCC9	NR1I2	
ALDH1A3	GSTM3	ABCD4	NR1I3	
ALDH1B1	GSTM4	ABCG2	NR3C1	
ALDH1L1	GSTM5	ABCG8	NR3C2	
ALDH2	GSTO1	ALD	NR5A2	
ALDH3A1	GSTO2	AQP1	PPARA	
ALDH3A2	GSTP1	AQP7	PPARD	
ALDH3B1	GSTT1	AQP9	PPARG	
ALDH3B2	GSTT2	ATP6V0C	PPARGC1A	
ALDH4A1	GSTT2B	ATP7A	PPARGC1B	
ALDH5A1	GSTZ1	ATP7B	PPRC1	
ALDH6A1	HNMT	KCNK9	PTGES3	
ALDH7A1	INMT	MARCKSL1	RARA	
ALDH8A1	MGST1	MDR/TAP	RARB	
ALDH9A1	MGST2	MRP	RARG	
AOC2	MGST3	MVP	RXRA	
AOC3	MPST	OABP	RXRB	
AOX1	NAA20	OATP2	RXRG	
BCHE	NAT1	SLC10A1	THRA	
CBR1	NAT2	SLC10A2	THRB	
CBR3	NNMT	SLC15A1	TRIP11	
CBR4	PNMT	SLC15A2	VDR	
CEL	PTGES	SLC16A1		
CES1	SAT1	SLC18A2		
CES2	SULT1A1	SLC19A1		
CES3	SULT1A2	SLC19A2		
CES4	SULT1A3	SLC19A3		
CES7	SULT1A4	SLC1A1		
CYP11A1	SULT1B1	SLC1A2		
CYP11B1	SULT1C2	SLC1A3		
CYP11B2	SULT1C3	SLC1A6		
CYP17A1	SULT1C4	SLC1A7		
CYP19A1	SULT1E1	SLC21A5		
CYP1A1	SULT2A1	SLC22A1		
CYP1A2	SULT2B1	SLC22A11		
CYP1B1	SULT4A1	SLC22A12		
CYP20A1	SULT6B1	SLC22A16		
CYP21A2	TPMT	SLC22A2		
CYP24A1	TST	SLC22A3		
CYP26A1	UGT1A1	SLC22A4		
CYP26B1	UGT1A10	SLC22A5		
CYP26C1	UGT1A3	SLC22A6		
CYP27A1	UGT1A4	SLC22A7		
CYP27B1	UGT1A5	SLC22A8		
CYP27C1	UGT1A6	SLC22A9		
CYP2A13	UGT1A7	SLC25A13		
CYP2A6	UGT1A8	SLC28A1		
CYP2A7	UGT1A9	SLC28A2		
CYP2B6	UGT2A1	SLC28A3		
CYP2C18	UGT2A3	SLC29A1		
CYP2C19	UGT2B10	SLC29A2		
CYP2C8	UGT2B11	SLC29A3		
CYP2C9	UGT2B15	SLC29A4		
CYP2D6	UGT2B17	SLC2A1		
CYP2E1	UGT2B28	SLC31A1		
CYP2F1	UGT2B4	SLC38A1		
CYP2J2	UGT2B7	SLC38A2		
CYP2R1	UGT3A1	SLC38A5		
CYP2S1	UGT3A2	SLC3A1		
CYP2U1		SLC3A2		
CYP2W1		SLC47A1		
CYP39A1		SLC47A2		
CYP3A4		SLC5A4		
CYP3A43		SLC6A3		
CYP3A5		SLC6A4		
CYP3A7		SLC7A11		
CYP46A1		SLC7A5		
CYP4A11		SLC7A6		
CYP4A22		SLC7A7		
CYP4B1		SLC7A8		
CYP4F11		SLCO1A2		
CYP4F12		SLCO1B1		
CYP4F2		SLCO1B3		
CYP4F22		SLCO1C1		
CYP4F3		SLCO2A1		
CYP4F8		SLCO2B1		
CYP4V2		SLCO3A1		
CYP4X1		SLCO4A1		
CYP4Z1		SLCO4C1		
CYP51A1		SLCO5A1		
CYP7A1		SLCO6A1		
CYP7B1		TAP1		
CYP8B1		TAP2		
DHRS2		VDAC2		
DHRS4		VDAC3		
DHRS9				
DPYD				
EPHX1				
EPHX2				
ESD				
FMO1				
FMO2				
FMO3				
FMO4				
FMO5				
HSD17B10				
KCNAB1				
KCNAB2				
KCNAB3				
KDM1A				
KDM1B				
MAOA				
MAOB				
NQO1				
NQO2				
PAOX				
PON1				
PON2				
PON3				
PTGIS				
PTGS1				
PTGS2				
SPR				
SUOX				
TBXAS1				
UCHL1				
UCHL3				
XDH				

**Table A2:** **Putative miRNAs associated with SNPs in the 3′-UTR region**.

Gene	Classification	SNP	Tissue	Putative miRNAs		
				microSNiPer	PolymiRTs	Overlap
ALDH16A1	Phase I	rs1055637	Liver	hsa-miR-4265	hsa-miR-3151	hsa-miR-4669
				hsa-miR-1231	hsa-miR-4447	
				hsa-miR-3120-5p	hsa-miR-4472	
				hsa-miR-4322	**hsa-miR-491-5p**	
				hsa-miR-4669	hsa-miR-132-5p	
				hsa-miR-4726-3p	hsa-miR-4669	
CYP2E1	Phase I	rs2480256	Liver	**hsa-miR-570**	**hsa-miR-570-3p**	**hsa-miR-570-3p**
CYP2E1	Phase I	rs2480257	Liver	hsa-miR-4762-5p	hsa-miR-5582-3p	
					**hsa-miR-570-3p**	
CYP2U1	Phase I	rs8727	Liver	**hsa-miR-549**	**hsa-miR-549**	**hsa-miR-549**
				hsa-miR-125b-2*		
CYP3A5	Phase I	rs15524	Liver	hsa-miR-562	**hsa-miR-500a-5p**	**hsa-miR-500a-5p**
				**hsa-miR-501-5p**	hsa-miR-5680	
				hsa-miR-500b		
				**hsa-miR-500a**		
				hsa-miR-4668-3p		
				hsa-miR-3973		
				**hsa-miR-362-5p**		
CYP3A7	Phase I	rs10211	Liver	N/A	**hsa-miR-125a-5p**	
					**hsa-miR-125b-5p**	
					hsa-miR-345-3p	
					hsa-miR-3920	
					hsa-miR-4319	
					hsa-miR-4732-3p	
					hsa-miR-670	
EPHX2	Phase I	rs1042032	Brain	hsa-miR-4476	hsa-miR-183-5p	hsa-miR-2392
				hsa-miR-4533	hsa-miR-2392	hsa-miR-183-5p
				hsa-miR-2392		
				**hsa-miR-432***		
				hsa-miR-761		
				hsa-miR-183		
				hsa-miR-3665		
				hsa-miR-32390		
EPHX2	Phase I	rs1042064	Brain	**hsa-miR-31**	hsa-miR-4696	hsa-miR-4696
				**hsa-miR-576-3p**		
				**hsa-miR-22**		
				hsa-miR-4696		
GSTM3	Phase II	rs1109138	Brain	hsa-miR-4766-3p	N/A	
				hsa-miR-2964a-3p		
				**hsa-let-7i***		
GSTM3	Phase II	rs1537236	Brain	hsa-miR-4762-5p	**hsa-miR-182-5p**	hsa-miR-4470
				hsa-miR-4470	hsa-miR-4470	
GSTM3	Phase II	rs1537235	Brain	hsa-miR-4790-3p	**hsa-miR-409-5p**	
GSTM3	Phase II	rs3814309	Brain	hsa-miR-4421	hsa-miR-3130-3p	
				hsa-miR-3182	hsa-miR-4793-3p	hsa-miR-4793-3p
				hsa-miR-1237		
				**hsa-miR-486-5p**		
				hsa-miR-4793-3p		
				hsa-miR-3120-5p		
				hsa-miR-4527		
				**hsa-miR-29b**		
GSTM5	Phase II	rs11807	Liver	hsa-miR-1202	N/A	
				hsa-miR-1227		
				hsa-miR-1973		
MGST3	Phase II	rs8133	Liver	hsa-miR-875-3p	**hsa-miR-582-3p**	**hsa-miR-582-3p**
				**hsa-miR-582-3p**	hsa-miR-875-3p	hsa-miR-875-3p
				hsa-miR-4698	hsa-miR-224-3p	hsa-miR-3688-3p
				hsa-miR-4694-3p	hsa-miR-3688-3p	hsa-miR-4694-3p
				hsa-miR-4495	hsa-miR-4694-3p	
				hsa-miR-411*	hsa-miR-522-3p	
				hsa-miR-3688-3p		
ATP7B	Phase III	rs928169	Liver	hsa-miR-4734	hsa-miR-4447	hsa-miR-4472
				hsa-miR-4430	hsa-miR-4472	hsa-miR-4481
				hsa-miR-4481	hsa-miR-4481	hsa-miR-4745-5p
				hsa-miR-4472	hsa-miR-4745-5p	hsa-miR-4785
				hsa-miR-3652	hsa-miR-4785	
				hsa-miR-3135b	hsa-miR-4787-5p	
				hsa-miR-4745-5p		
				hsa-miR-3944-3p		
				hsa-miR-1275		
				**hsa-miR-491-5p**		
				hsa-miR-4446-3p		
				hsa-miR-4498		
				hsa-miR-194*		
				**hsa-miR-122**		
				hsa-miR-4734		
				hsa-miR-4430		
				hsa-miR-3652		
				hsa-miR-4309		
				hsa-miR-4785		
				hsa-miR-3198		
				hsa-miR-1298		
SLC31A1	Phase III	rs10759637	Liver	hsa-miR-4448	hsa-miR-3672	
				hsa-miR-3119	hsa-miR-4524a-3p	
				hsa-miR-4461		
TAP2	Phase III	rs13501	Brain	hsa-miR-3198	hsa-miR-1289	hsa-miR-1289
				hsa-miR-1289	hsa-miR-3198	hsa-miR-3198
				hsa-miR-4309	hsa-miR-4294	hsa-miR-4309
				hsa-miR-3127-5p	hsa-miR-4309	
					hsa-miR-5702	
TAP2	Phase III	rs17034	Brain	hsa-miR-4772-3p	**hsa-miR-1271-3p**	
					hsa-miR-4763-5p	
					hsa-miR-550a-3-5p	
					hsa-miR-550a-5p	
					hsa-miR-4327	
					hsa-miR-636	
TAP2	Phase III	rs241451	Brain	hsa-miR-1260	hsa-miR-4684-5p	hsa-miR-4684-5p
				hsa-miR-4758-3p		
				hsa-miR-4684-5p		
TAP2	Phase III	rs241452	Brain	hsa-miR-1206	hsa-miR-1206	hsa-miR-1206
				**hsa-miR-1**		
				hsa-miR-4789-5p		
TAP2	Phase III	rs241453	Brain	hsa-miR-4298	hsa-miR-1302	hsa-miR-1302
				hsa-miR-1302	hsa-miR-4298	hsa-miR-4298
TAP2	Phase III	rs241454	Brain	hsa-miR-4476	hsa-miR-4476	hsa-miR-4476
				hsa-miR-4779	hsa-miR-4533	hsa-miR-4779
					hsa-miR-3173-3p	
					hsa-miR-4779	
TAP2	Phase III	rs241455	Brain	**hsa-miR-130a***	hsa-miR-2116-3p	**hsa-miR-130a-5p**
				**hsa-miR-323-3p**	**hsa-miR-130a-5p**	
					**hsa-miR-23a-3p**	
					**hsa-miR-23b-3p**	
					hsa-miR-23c	
					hsa-miR-3680-5p	
					hsa-miR-4798-3p	
TAP2	Phase III	rs241456	Brain	hsa-miR-3940-5p	hsa-miR-2110	hsa-miR-4450
				hsa-miR-4507	hsa-miR-3150a-3p	
				**hsa-miR-92a-1***	hsa-miR-4450	
				hsa-miR-4450	**hsa-miR-450a-3p**	
					**hsa-miR-1270**	
					hsa-miR-3676-5p	
					hsa-miR-4531	
					hsa-miR-4683	
					hsa-miR-620	
TAP2	Phase III	rs2857101	Brain	hsa-miR-944	**hsa-miR-126-5p**	hsa-miR-944
				hsa-miR-4795-3p	hsa-miR-4795-3p	hsa-miR-4795-3p
				hsa-miR-183*	hsa-miR-944	
UGT2A1	Phase II	rs4148312	Liver	hsa-miR-548t	hsa-miR-3662	hsa-miR-3662
				hsa-miR-548ah	**hsa-miR-548c-3p**	hsa-miR-3609
				hsa-miR-3662	hsa-miR-3609	hsa-miR-548ah-5p
				hsa-miR-3646	hsa-miR-548ah-5p	hsa-miR-548t-5p
				hsa-miR-3609	**hsa-miR-548n**	
				**hsa-miR-340**	hsa-miR-548t-5p	
				**hsa-miR-1245**		
				**hsa-miR-106a**		
ARNT	Nuclear receptors	rs11552229	Liver	hsa-miR-4716-5p	hsa-miR-4717-3p	

*The miRs expressed in the tissue where the eQTL was identified are highlighted in bold*.
